# A Closer Look into White Adipose Tissue Biology and the Molecular Regulation of Stem Cell Commitment and Differentiation

**DOI:** 10.3390/genes15081017

**Published:** 2024-08-02

**Authors:** Presley D. Dowker-Key, Praveen Kumar Jadi, Nicholas B. Gill, Katelin N. Hubbard, Ahmed Elshaarrawi, Naba D. Alfatlawy, Ahmed Bettaieb

**Affiliations:** 1 Department of Nutrition, University of Tennessee Knoxville, Knoxville, TN 37996-0840, USA; 2 Graduate School of Genome Science and Technology, University of Tennessee, Knoxville, TN 37996-0840, USA; 3 Department of Biochemistry, Cellular and Molecular Biology, University of Tennessee, Knoxville, TN 37996-0840, USA

**Keywords:** white adipose tissue, adipogenesis, stem commitment and differentiation, mitotic clonal expansion, metabolism

## Abstract

White adipose tissue (WAT) makes up about 20–25% of total body mass in healthy individuals and is crucial for regulating various metabolic processes, including energy metabolism, endocrine function, immunity, and reproduction. In adipose tissue research, “adipogenesis” is commonly used to refer to the process of adipocyte formation, spanning from stem cell commitment to the development of mature, functional adipocytes. Although, this term should encompass a wide range of processes beyond commitment and differentiation, to also include other stages of adipose tissue development such as hypertrophy, hyperplasia, angiogenesis, macrophage infiltration, polarization, etc.… collectively, referred to herein as the adipogenic cycle. The term “differentiation”, conversely, should only be used to refer to the process by which committed stem cells progress through distinct phases of subsequent differentiation. Recognizing this distinction is essential for accurately interpreting research findings on the mechanisms and stages of adipose tissue development and function. In this review, we focus on the molecular regulation of white adipose tissue development, from commitment to terminal differentiation, and examine key functional aspects of WAT that are crucial for normal physiology and systemic metabolic homeostasis.

## 1. Introduction: An Overview of Adipose Tissue Biology and Function

Adipose tissue (AT) is a complex and multifunctional organ found in mammals and several non-mammalian species [1,2]. In mammals, AT is organized into distinct regions known as fat depots and distributed throughout various parts of the body [1,3,4]. AT is typically classified into two primary depots based on anatomical location: subcutaneous (beneath the skin) and visceral (within or surrounding body cavities) [1,3,4]. In small mammals, such as rodents, which are commonly used as preclinical models for obesity research [5], anterior subcutaneous fat includes the interscapular, subscapular, superficial cervical, and axillo-thoracic regions, while posterior subcutaneous fat is found in the inguinal, gluteal, and dorso-lumbar areas. Visceral adipose tissue is located within the abdominal cavities and thorax of these species [6,7,8]. Comparatively, in humans, white adipose tissue (WAT) is also distributed across the body, encompassing both subcutaneous (intramuscular, abdominal, gluteofemoral) and visceral (mesenteric, gonadal, pericardial, retroperitoneal, omental) depots [3,9]. Besides these primary depots, brown adipose tissue (BAT) is a functionally distinct entity. In rodents, BAT is present in the cervical, perirenal, supraclavicular, paravertebral, axillary, and inter-/intrascapular depots [1,3,6]. In humans, BAT is prominent during the infancy stage and is located in the intrascapular (major depot), cervical, and perirenal regions; then, it later extends towards the trunk and limbs [3,10]. BAT is crucial for maintaining core body temperature during early development and was previously thought to be overall insignificant in adulthood. However, later evidence has documented active BAT depots in adult humans, in both visceral and subcutaneous locations [3,4,10]. Visceral brown fat is located perivascularly, periviscally, or around solid organs, while subcutaneous brown fat is found beneath the clavicles, within the axilla and anterior abdominal wall, and between the supraclavicular fossa and anterior cervical muscles [10,11]. The phenotypic traits of white and brown adipose depots are primarily determined by the ratio of brown to white adipocytes [12,13]. However, the phenomenon of “browning” or “beiging”, wherein white adipocytes convert to brown-like adipocytes (also known as beige or brite), highlights the plasticity and functional potential of adipose tissue [12,14].

Additionally, the literature acknowledges two less-characterized types of adipose tissue: pink and yellow adipose tissue [15]. Although their clinical relevance remains elusive, pink adipocytes (PATs) in rodents, mice in particular, arise through the process of “pinking”, wherein white adipocytes in the mammary glands transform into milk-producing epithelial cells during pregnancy and lactation [16,17]. Yellow adipose tissue (YAT) is described as a fat pad within the bone marrow, exhibiting characteristics of both WAT and BAT and speculated to play a role in systemic energy metabolism, as well a plethora of human diseases [18,19,20]. 

The distinct characteristics of these fat depots, especially between BAT and WAT, coupled with emerging evidence demonstrating the potential to modulate the fate of adipogenesis by promoting the browning of WAT for example, are generating significant interest due to their profound implications in health and metabolic regulation [13,21]. As such, understanding the fundamentals of adipose biology offers promising avenues for developing therapeutic strategies aimed at combating obesity and related metabolic disorders. Notably, the ability to modulate adipocyte phenotype and function could provide novel interventions for enhancing energy expenditure and improving metabolic outcomes in individuals with obesity and associated diseases.

It is worth noting that the functional heterogeneity of adipose tissue extends beyond anatomical distribution. Indeed, WAT and BAT display distinct differences in cell population, function, and morphology that are reflective of their specialized roles in energy metabolism and endocrine functions, in particular [4,13,22]. White adipocytes are distinguished by a large, unilocular lipid droplet, which occupies most of the cell volume, and a peripherally compressed nucleus, indicative of their primary function in long-term energy storage. These cells possess low mitochondrial content, further underscoring their role in storing excess energy as triglycerides and releasing free fatty acids during periods of energy demand [23]. In contrast, brown adipocytes contain multiple smaller, multilocular lipid droplets, which facilitate rapid lipid mobilization [22,23]. These cells are densely packed with mitochondria containing high levels of the hallmark marker, uncoupling protein 1 (UCP1) [23]. UCP1 is critical for non-shivering thermogenesis, as it uncouples oxidative phosphorylation in the mitochondria, allowing for heat production instead of ATP synthesis [24,25]. This thermogenic capability of BAT plays a vital role in maintaining body temperature and energy expenditure, particularly in response to cold exposure [24,26]. Likewise, beige adipocytes, which reside within WAT depots, share phenotypic similarities with white adipocytes but possess the inducible capacity to express brown-like thermogenic markers upon exposure to specific stimuli [27].

Advances in research on obesity and its associated diseases have led to a paradigm shift in the understanding of WAT, transforming its perception from a passive lipid repository to a complex endocrine organ critically involved in systemic energy homeostasis [28,29]. Characterized by unilocular adipocytes with a size range of 20–100 µm, and influenced by factors such as body mass index, diabetic status, and anatomical location, WAT exhibits remarkable plasticity [29] and plays key roles in energy homeostasis through the regulation of lipogenesis and lipolysis. Lipids stored in WAT as triacylglycerol (TAG) are replenished via dietary intake or endogenous production through de novo lipogenesis (DNL). WAT also functions as an endocrine organ, secreting a large number of adipokines with pleiotropic effects on appetite, energy metabolism, insulin sensitivity, inflammation, and other physiological processes. In addition to these roles, WAT provides essential mechanical support, thermal insulation, immune responses, wound healing, and many other physiological processes [29]. Therefore, the dysregulation of WAT function, such as what is observed in obesity for example, contributes to the pathogenesis of several metabolic disorders, emphasizing the need for a comprehensive understanding of WAT biology for therapeutic interventions.

Similar to WAT, BAT also exhibits a remarkable plasticity with significant implications for metabolic health and disease, adapting its cellularity and functionality in response to various physiological, nutritional, and environmental stimuli [30]. BAT plasticity plays also a pivotal role in mediating its thermogenic potential and contribution to energy balance, particularly in response to cold exposure and dietary factors. The molecular mechanisms underlying BAT plasticity involve a complex network of transcriptional regulators and signaling pathways. Key transcription factors, such as peroxisome proliferator-activated receptor gamma (PPARγ), PR domain containing 16 (PRDM16), and PPARγ coactivator-1 alpha (PGC-1α), integrate signals from hormonal, neural, and nutritional inputs to orchestrate the differentiation and activation of brown and beige adipocytes and modulate the expression of thermogenic genes, UCP1 in particular. For instance, β-adrenergic signaling induction in BAT, upon cold exposure, activates its thermogenic function through the upregulation of UCP1 via the p38 mitogen-activated protein kinase (MAPK)/Cyclic AMP (cAMP) axis [31]. β-adrenergic stimulation in BAT also regulates the activity of other genes involved in lipid and glucose metabolism as well as the uptake and oxidation of other metabolic substrates [32,33,34]. In addition to inhibiting the lysosomal degradation of UCP1, the β-adrenergic signaling cascade enhances UCP1’s activity by promoting the binding of free fatty acids (FFAs) to UCP1 [35,36] and/or facilitating the release of purine nucleotides from UCP1 [32,33,37,38]. This results in a UCP1-dependent thermogenesis characterized by the dissipation of energy in the form of heat, while also promoting fatty acid oxidation [39]. Consequently, BAT activity has been shown to be inversely associated with obesity and related metabolic disorders in humans and rodent models. However, impaired BAT function is directly correlated with various metabolic diseases [40,41,42,43,44,45]. 

The precise mechanisms underlying the association between BAT mass/activity and metabolic health remain to be fully elucidated, as some studies have challenged the inverse relationship between BAT volume or activity and obesity in humans [46,47]. However, it is noteworthy to indicate that, while BAT’s robust capacity for triglyceride and glucose utilization contributes significantly to its metabolic benefits, the observed metabolic effects following the transplantation of committed brown preadipocytes suggest that additional factors are at play [48]. Emerging evidence indicates that BAT actively secretes a plethora of endocrine modulators, termed “batokines”, which confer several metabolic benefits. Among these, fibroblast growth factor 21 (FGF21) is particularly significant, as its release following thermogenic activation may mediate systemic metabolic improvements [48,49]. 

It is also worth noting that, while the larger body of evidence supports the clinical benefits associated with increased BAT volume and activity as well as the browning of WAT in several metabolic diseases, the impact on cardiometabolic health remains complex. For example, even though a preponderance of evidence supports a cardioprotective role for BAT, emerging data indicate potential adverse effects in certain circumstances. For instance, a recent study found direct correlations between BAT volume and activity, homocysteine levels, and markers of liver damage in a Betaine-homocysteine S-methyltransferase (BHMT)-knockout (KO) mouse model, indicating increased cardiometabolic risk despite improvements in energy expenditure, glucose uptake, and overall glycemic control [50]. Similarly, under certain pathological conditions, such as severe illness [51,52], dermal burn-induced hypermetabolism [53,54], cancer-related cachexia [55,56], and sepsis [57,58], increased BAT activity may be unfavorable. Additionally, strategies designed to enhance BAT mass and function, especially those that involve increasing sympathetic nervous system (SNS) activation of BAT, may pose significant health risks. Chronic SNS activation can lead to elevated blood pressure, cardiovascular contraction, and heartbeat enhancement and acceleration [59,60]. This underscores the need for a cautious and thorough understanding of adipose tissue biology, encompassing both BAT and WAT, before implementing such therapeutic strategies. 

Ensuring that interventions targeting adipose tissue do not inadvertently compromise whole-body homeostasis is and will remain essential. It should be recognized that while the metabolic and clinical benefits of healthy BAT function have since garnered extensive review, WAT has received comparatively less attention. This review aims to address that gap and provide a thorough examination of WAT within the framework of adipose tissue biology at the cellular levels.

## 2. WAT Development

### 2.1. Cellular Hierarchy

All complex organisms, including humans, abide by the biological levels of organization that begin with a single cell to an entire organ system. Therefore, to gain insight into adipose tissues’ contribution to human health and disease, it is necessary to understand not only the development of adipocytes, the main cell type of the organ, but also the contribution of the stromal vascular fraction (SVF) and, together, how they orchestrate the functions of adipose tissue. Although the adipose organ comes in a variety of ‘hues’, the one common denominator that relates all types of adipose tissue is lipid content. In WAT, these adipocytes are specially equipped for the storage of TAG during times of positive energy intake [61]. 

However, prior to white adipocytes’ maturity and energy storage potential, immature adipocytes, regarded as ‘adipose progenitor (AP)/adipose precursor cells (APCs)’, ‘preadipocytes’, ‘adipose-derived stromal/adipose stem/adipose mesenchymal stem cells’ (ADSCs, ASCs, Ad-MSCs), or adipose stem and progenitor cells (ASPCs), are matured through a meticulous orchestration known as adipocyte differentiation [62,63,64]. Notably, immature adipocytes reside in the already heterogenous SVF of AT in addition to occupancy of mature adipocytes [65,66,67,68]. Both the existing and emerging literature continue to loosely handle these terms and refer to them either synonymously or collectively. This inconsistency leaves for an open interpretation and, consequently, confusion. Therefore, a hierarchical system to appropriately sort these definitions is warranted. 

It is widely acknowledged that adipocytes are derived from a mesodermal origin [69]. However, others have demonstrated the emergence of adipocytes outside this traditional germ layer and provide evidence that the neuroectoderm also produces adipocytes [70,71]. This latter evidence is supported by studies demonstrating that both layers are able to source mesenchymal stem cells (MSCs) [72,73,74]. However, it should be noted that The International Society for Cell & Gene Therapy (ISCT^®^) Mesenchymal Stromal Cell (ISCT MSC) committee stands firm on the notion that the term mesenchymal “stem” cells ≠ mesenchymal “stromal” cells. Minimally, stromal cells are defined by several criteria such as: (i) secretory and migratory ability, (ii) capacity to modulate the immune response, (iii) specified cell surface markers, and (iv) multipotency [75,76,77]. Despite efforts to resolve this issue, the field has yet to fully adopt this standard and, therefore, in this review, we have chosen to use the terminology of the paper being cited.

By context, ADSCs, ASCs, and Ad-MSCs indicate populations of cells that have the ability to differentiate into cell populations other than adipocytes including chondrocytes (cartilage), osteoblasts (bone), and myoblasts (muscle) [78,79]. The “stemness” of these cell populations has been traditionally demonstrated through the identification of various cell surface markers and transcriptional profiling (extensively reviewed elsewhere) [80,81]. Briefly, early attempts to sort the SVF have included the use of the fluorescence-activated cell sorting (FACS) system, culminating in the isolation and identification of a subpopulation of SVF-resident cells capable of establishing WAT in vivo. Additionally, this subpopulation was shown to also give rise to other mesenchymal derivatives in vitro [65]. In a later study, the previously identified SVF cell population, denoted as Lin−:CD29+:CD34+: Sca-1+:CD24+, was then committed to the adipogenic lineage by the loss of CD24 expression, thus becoming Lin−:CD29+:CD34+:Sca-1+:CD24− and expressing markers of late-stage adipocyte differentiation [65,66]. Single-cell molecular approaches, such as single-cell RNA sequencing (scRNA-seq), have also been utilized to deconvolute the molecular signatures of these subtypes [82]. An integrative meta-analysis revealed that Cd34, Cd29, and platelet-derived growth factor receptor alpha (Pdgfra) were validated reliably across all ASPC subpopulations while surface markers Cd24 and Pdgrfb were inconsistently present, supporting previous FACS observations [82]. Additionally, using scRNA-seq analyses, Merrick and colleagues reported that a subgroup of progenitor cells, marked by dipeptidyl peptidase–4 (DPP4+), exhibit a demeanor reminiscent of mesenchymal cells and reside within a surrounding niche they refer to as the reticular interstitium. However, committed cells were found to express preadipocyte factor 1 (Pref-1) and intercellular adhesion molecule–1 (ICAM1), as well as several major adipogenic gene identifiers such as fatty acid binding protein 4 (*Fabp4*) and *Pparg* [83]. Moreover, a novel subpopulation from the SVF of WAT was reported to possibly precede committed preadipocytes and suggestibly acts as a transitional, yet necessary, pool of precursors the authors considered as ‘pro-preadipocytes’ (ICAM1+ CD44 high Noct-expressing) [84]. Fortunately, the higher resolution of single-cell approaches is expected to further delineate this population of immature precursors and, in turn, optimize the field’s ability to appropriately isolate and investigate the dynamics of adipocyte development.

### 2.2. Commitment

**Figure 1:**
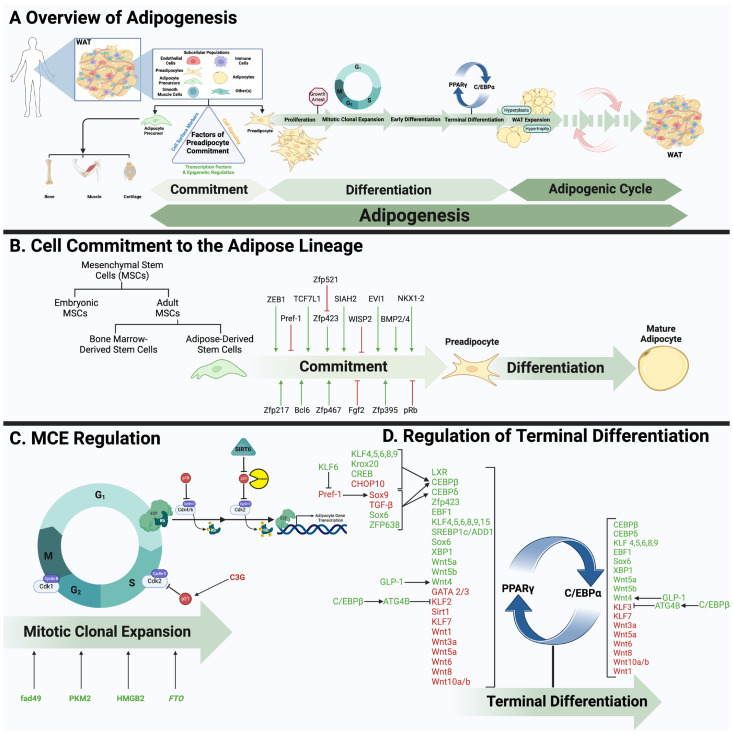
Molecular controls and regulations of adipocyte development. (**A**) WAT is found throughout the human body and congregates in specific sites referred to as fat depots. The two major depots, subcutaneous (SAT) and visceral (VAT), both serve distinct metabolic and nonmetabolic roles in the body. Although WAT depots are heterogenous, adipocytes account for more than 90% of WAT volume [67], with the remaining collectively known as the stromal vascular fraction (SVF). The SVF is composed of various cell types that contribute to WAT’s growth, repair, renewal, and functioning. Importantly, a subpopulation of immature adipocytes resides in this fraction, awaiting the appropriate cues to undergo maturation. (**A**) In a highly strategic manner, an adipose precursor cell is (1) committed to the adipogenic lineage and then sequentially undergoes (2) proliferation, (3) clonal expansion (MCE), (4) early- and (5) late-stage differentiation. (6) Adipocyte expansion via hyperplasia and/or hypertrophy, along with processes such as cell turnover, angiogenesis and macrophage infiltration attribute to the establishment of mature adipocytes and WAT (All together referred to as the adipogenic cycle). (**B**,**C**) Various molecular regulators have been shown to either promote or inhibit stem/stromal cell commitment as well as MCE, both critical phases of adipocyte differentiation. (**D**) Terminal differentiation is largely characterized by PPARγ and C/EBPα, notably the master transcription factors. Equally as important is their maintenance by upstream modulators that either promote or inhibit the master transcription factors. Figure created with BioRender.com.

Another consideration remains: ‘what’ causes cells with a multipotent propensity to commit to a single course trajectory, an adipocyte? Over the last few decades, various transcription factors and mechanisms have been revealed to either mark this commitment, direct it, or both (Table 1, Figure 1A,B). 3T3-L1 cells have served as a standard sub-clonal cell line to track and study the course of adipose tissue development. Notably, due to their fibroblastic morphology and pre-commitment to the adipocyte lineage, 3T3-L1 cells are commonly referred to as 3T3-L1 ‘preadipocytes’ [85,86,87,88]. 

One of the founding, and most classical, markers used to distinguish preadipocytes is Pref-1 or delta-like homolog–1 (Dlk1) on account of its transient, from high to ‘no’, expression across the course of 3T3-L1 cell differentiation [89,90,91]. However, Pref-1 exceeds well beyond its esthetic and has been shown to participate in mesenchymal cell commitment and regulate adipocyte differentiation [92,93]. Wang and Su found that induction of SRY-box transcription factor 9 (Sox9), a factor expressed following Pref-1’s activation of ERK1/2 signaling, promotes mesenchymal cell preference towards the chondrogenic lineage yet does not yield in the maturation of chondrocytes, adipocytes, or osteocytes. That is, not until Sox9 directly binds to CCAAT-enhancer-binding protein (C/EBP) β and C/EBPδ promoter regions and suppresses their activity when mediating Pref-1’s function as an inhibitor of adipocyte differentiation [92,93]. Consequently, inhibition of Sox9 appears essential for adipocyte differentiation. This notion is supported by studies demonstrating that upregulation of Pref-1, an upstream regulator of Sox9, suppresses, while conversely, Pref-1 suppression promotes adipocyte differentiation [89]. 

Although Pref-1 has been the field’s conventional preadipocyte marker [94], emerging evidence implicates it as a critical factor, among others, in both the commitment and differentiation stages of adipocyte development. Other factors include the RING-type E3 ubiquitin transferase SIAH2 [95,96], several cell cycle regulators such as transcription factor 7-like 1 (TCF7L1) [97] and retinoblastoma protein (pRb) [98,99], various zinc finger proteins (Zfp) including B-cell lymphoma 6 (Bcl6) [100], Evi1 [101,102,103], Zfp217 [104,105,106], Zfp395 [107], Zfp423 [108,109], Zfp467 [110,111], Zfp521 [112,113], and Zinc finger E-box-binding homeobox 1 (ZEB1) [114], as well as the transcriptional regulator NK1 Homeobox 2 (NKX1-2) [115] (Table 1, Figure 1B). These factors have been shown to play crucial roles in precursor cell commitment to the adipocyte lineage, the differentiation process, and maintenance of differentiated adipocytes through diverse mechanisms [100,116,117]. For instance, ectopic expression of Zfp423 has been shown to upregulate the peroxisome proliferator-activated receptor gamma 2 (PPARγ2), leading to significant adipocyte differentiation in non-adipogenic NIH 3T3 cells. It was initially suggested that Zfp423 mediates this pro-adipogenic response partly by enhancing SMAD/BMP signaling [108,109]. However, recent studies have revealed that preadipocytes overexpressing a SMAD-binding domain-deficient mutant of Zfp423 can still differentiate into fully mature adipocytes [108]. These findings indicate that Zfp423 can promote adipogenic differentiation independently of the SMAD/BMP axis, suggesting a more complex role in adipocyte commitment [116,118]. 

Likewise, findings from the ZEB1 studies have also led to controversial findings. Meanwhile, initial studies have reported that ZEB1 haploinsufficiency caused excessive fat accumulation in female mice during the early phases of high-fat feeding and acquisition of adipose tissue [119]. In contrast, later studies by Gubelmann and colleagues, using 3T3-L1 preadipocytes, suggested that ZEB1 acts as a positive regulator in the stages of adipocyte differentiation. The study demonstrated that ZEB1 overexpression increased *Pparg2* and *Cebpa* expression, while ZEB1 knockdown altered the expression of several key transcription factors, including *Cebpa* and *Id4* [114]. The precise molecular mechanisms underlying ZEB1’s role in adipocyte differentiation remain to be fully elucidated. As such, further investigations are warranted as the complete ablation of the TCF8 gene, encoding the ZEB1 transcription factor, is perinatally lethal [119,120]. These findings also underscore the need for careful consideration when designing anti-obesity strategies that target this protein.

In addition to Zfp proteins, bone morphogenetic proteins (BMPs) have already been evaluated for their potential adipogenic properties. Tang and Lane demonstrated that BMP4 treatment of pluripotent C3H10T1/2 stem cells promoted their commitment to the adipose lineage. These cells subsequently differentiated into adipocytes, characterized by lipid accumulation and the expression of adipogenic markers including C/EBPα, PPARγ, and fatty acid-binding protein 4 (FABP4; a.k.a. aP2) [121]. Importantly, BMP4 has been demonstrated to disrupt the cytosolic WNT1 inducible signaling pathway protein 2 (WISP2)-Zfp423 complex, thereby liberating Zfp423 and subsequently activating PPARγ, ultimately inducing the differentiation of NIH 3T3 cells into mature adipocytes [122]. Additional members of the Zfp family have also been implicated in regulating adipocyte lineage commitment. Zfp521 inhibits adipogenic differentiation through its interaction with early B cell factor 1 (Ebf1), which sequesters Zfp423, preventing the activation of *Pparg* [113]. Conversely, Zfp467 promotes adipocyte differentiation over osteogenesis, a process potentially mediated by its modulation of Wnt signaling [110,111,123,124]. Aside from these prominent genes/families of genes, more recent investigations have led to the discovery of various other factors that regulate adipocyte commitment. For instance, the gene *Msi2*, which encodes for the RNA-binding protein Musashi 2, has been shown to govern the lineage commitment of bone marrow mesenchymal stem cells (BMSCs) by altering PPAR signaling. The authors report that when *Msi2* is knocked-out in mice, BMSCs commit to an adipogenic lineage due to a lift on the restriction of *Cebpα* translation, a critical maintenance factor of both PPARγ and terminal differentiation (thoroghly discussed later) [125]. Collectively, these factors and mechanisms (Table 1) not only mark or direct preadipocyte commitment but are also pertinent for supplying an adequate pool of progenitors to aid in adipose development and/or maintenance. 

### 2.3. Adipose Tissue Expansion

In humans, adipocytes can be detected as early as the intrauterine period [126] yet continue to expand throughout the stages of development into adolescents [127]. Interestingly, it has been shown that ~10% of adult human adipocytes are replenished annually to compensate for adipocyte losses due to cell death [128]. Therefore, the formation of adipose tissue seems to be just as relevant postnatally as it is during embryogenesis. Accordingly, APCs have been characterized based on their participation during either organogenesis (developmental APs) or later, such as in AT maintenance and homeostasis (adult APs). Several groups have pursued the identification of molecular signatures and interactions that distinguish the two populations [129,130]. For example, some studies propose that developmental, but not adult, APCs are sourced from PDGFRα+ cells, while adult APCs exhibit mural markers such as smooth muscle actin (SMA+) and Pdgfrβ+ [129,131,132]. 

In addition, several non-adipose tissues including the bone and skeletal muscle have been shown to contribute to the overall pool of progenitors that give rise to adipocytes. As aforementioned, a subset of bone-resident cells that commit to the adipogenic lineage, referred to as marrow adipose tissue, has been shown to contribute to poor age-related bone healing and hematopoietic regeneration [133]. Others have described subpopulations of muscle-derived cells to also possess adipogenic tendency. For example, PDGFRα+ mesenchymal progenitors derived from the muscle interstitium were shown to efficiently differentiate into adipocytes, exhibiting lipid accumulation and expressions of late-stage differentiation markers C/EBPα and PPARγ [134]. Additionally, a subset of muscle-resident cells, denoted as fibro/adipogenic progenitors (FAPs), have demonstrated adipogenic potential and have been implicated in muscle tissue regeneration in response to injury [135,136]. Therefore, these examples imply that characterizing progenitor niches and their variability is pertinent to understanding their specific roles in adipose tissue development. 

The adipose organ is known to expand by two modes, either by increasing in (1) cell size (hypertrophy) or (2) number (hyperplasia) [128,137]. Throughout the human lifespan, WAT largely accommodates for caloric flux. For instance, in response to positive caloric intake, the organ can utilize its expansion to support the increased demand for energy storage. Conversely, during times of fasting, WAT appropriately responds to this caloric deficit through the mobilization of energy storage [138]. While these processes are normal physiological responses to maintain energy balance, in states of chronic altered nutrition, including both over (obesity) and under (lipoatrophy) nutrition, the adipose organ is highly susceptible to the detriments of each extreme. For example, in overnutrition, pathologic expansion occurs when adipocytes reach and necessitate outside their storage capacity for lipids. Beyond this storage threshold, several pathological traits of adipocytes begin to emerge including hypoxia, fibrosis, pathological hypertrophy, and chronic inflammation [139,140]. However, not all adipose tissue expansion is detrimental. In fact, several factors that determine healthy vs. unhealthy expansion have been linked to adipose type and location. 

In humans, adipose tissue is largely sub-categorized as either subcutaneous (SAT) or visceral (VAT). SAT is located under the skin while VAT is concentrated intra-abdominally and lines internal organs. VAT, by its association to metabolic consequence, is typically referenced as “bad” fat, while SAT accumulation is presented as a healthier, more metabolically protective type [141,142,143]. For example, one study demonstrated a significant correlation between VAT volume, VAT-to-SAT ratio, and glycemic control, independent of confounders such as BMI, dyslipidemia, and smoking [144]. Moreover, a Swedish study utilizing radiocarbon dating revealed that lipid turnover associated with unhealthy obesity might be depot-specific, with elevated visceral and/or reduced subcutaneous adipose tissue turnover linked to metabolic dysfunction [145]. In contrast, metabolically healthy obesity (MHO) describes individuals with obesity—in accordance with BMI metrics—that lack overt cardiometabolic disturbances. Studies have indicated that persons with MHO bear less visceral adiposity and increased subcutaneous stores in comparison to individuals with metabolically unhealthy obesity (MUO) [146,147,148]. Even in individuals with a normal BMI, risk of cardiovascular disease (CVD) incidence was shown to be higher in postmenopausal women when central adiposity was elevated and leg fat was reduced [149]. 

Another popular theme that underscores healthy vs. unhealthy expansion infers the concept of “how” adipose tissue expands—by number or by size. Large hypertrophic adipocytes are associated with compromised cellular function, metabolic dysregulation, and various pathologies [150]. For example, an early 2000s cross-sectional study proposed that an enlarged cell size of adipocytes can predict type 2 diabetes mellitus (T2DM) in addition to, yet independent of, insulin resistance (IR) or obesity [151]. Furthermore, it was demonstrated that hypertrophic epicardial adiposity shares a direct relationship with the severity of coronary artery disease [152]. Supportively, a recent transcriptome analysis concluded that several gene expressions related to lipid metabolism and mitochondrial functioning are markedly altered in hypertrophic human adipose tissue [153]. Therefore, healthy AT expansion is prominently associated with hyperplasia, as it is implied that hyperplasia helps preserve the metabolic function of AT [154,155]. For instance, transgenic mice overexpressing C/EBPβ, a key adipogenic transcription factor, on a high-fat diet exhibited increased adipocyte number rather than size, and were protected from diet-induced metabolic disturbances [156,157]. Conversely, adipose tissue from diabetic bariatric surgery patients displayed reduced preadipocyte numbers and increased fibrosis, conditions often linked to adipocyte hypertrophy [140]. However, a subsequent study suggested that type 2 diabetes-related adipose tissue hypertrophy and impaired differentiation might be attributed to progenitor cell senescence rather than a lack of precursor cell quantity [158]. 

However, and more critically, healthy obesity is a rather dissonant concept regardless of its expansion modality or metabolic consequence—and/or lack thereof. In fact, both clinical and preclinical studies have shown that hypertrophic expansion merely precedes hyperplastic growth during overnutrition [159,160], and weight relapse in rodent studies is supported by hyperplasia that manifest provisions for hypertrophy [161,162,163]. Hence, both processes may reciprocally regulate each other to promote disease progression. In addition, it is well established that excess body weight impacts a variety of non-metabolic parameters, for example, joints, physical mobility, and respiration as well as intangible factors such as psychosocial health [164,165]. Therefore, healthy obesity may be “metabolically favorable” in comparison to MUO, yet still perturb the dynamic of overall health and wellbeing.

### 2.4. Adipose Stromal Cells

As previously stated, within the AT’s SVF reside many non-adipocytes cell types, including the previously described group of heterogeneous precursor cells (Section 2.1), in addition to both immune and vascular cells. Interestingly, adipocytes have been shown to engage in crosstalk with many components of the SVF that direct various processes such as cell differentiation or inflammation. For example, interleukin-1β (IL-1β), a major cytokine derived from macrophages as well as other cells, was demonstrated to be markedly increased in primary human adipocytes exposed to macrophage-conditioned medium, concomitant with an impairment of insulin sensitivity. These effects were shown to be reduced upon the neutralization of IL-1β activity and, furthermore, IL-1β depletion restored the expression of major proteins involved in the insulin signaling pathway, including insulin receptor substrate 1 (IRS1), glucose transporter 4 (GLUT4), and pAkt [166]. 

IL-1β, as well as other interleukin-1 family members, have also been implicated in the maintenance of adipocyte differentiation. Inhibition of lipid accumulation and decreased expression of PPARγ and C/EBPα were observed in 3T3-F442A cultures treated with IL-1β. Comparable results were also obtained using primary human adipocytes [167]. IL-1F6, a major secreted cytokine of resident AT macrophages, was also shown to negatively regulate adipocyte differentiation by downregulating the expression of PPARγ [168]. Similarly, adipocytes have also been shown to exchange cues with endothelial cells and contribute to the regulation of lipid and glucose metabolism as well as WAT development [169,170]. Hammel and Bellas demonstrated that adipocytes co-cultured with human umbilical vein endothelial cells (HUVECs) at 1:1 and 1:3 ratios hinder the expression of *PPARG* [170]. Additionally, another subpopulation of stromal cells, referred to as adipogenesis-regulatory (Areg) cells, have been shown to suppress adipocyte formation via paracrine crosstalk both in vitro and in vivo [171]. In summary, although obesity research is mainly focused on the adipocyte itself, examining the interrelationship between adipocytes and their supporting cells warrants additional consideration. 

**Table 1:** Select Markers and Regulators of Preadipocyte Commitment and Differentiation.

Markers/Regulators	Overall Effect on WAT Development	Contribution	Commitment vs. Differentiation	Reference
Bcl6	Promotes	Regulator	Commitment	[100]
BMP2/4	Promotes	Regulator	Commitment	[121,172]
EVI1	Promotes	Regulator	Commitment, Differentiation	[101,102,103]
Fgf2	Inhibits	Regulator	Commitment, Differentiation	[173]
NKX1-2	Promotes	Regulator	Commitment, Differentiation	[115]
Pref-1	Inhibits	Marker, Regulator	Commitment, Differentiation	[91]
		Regulator	Commitment	[93]
pRb	Inhibits	Regulator	Commitment, Differentiation	[98,99,174]
SIAH2	Promotes	Regulator	Commitment	[95,96]
TCF7L1	Promotes	Regulator	Commitment	[97]
ZEB1	Promotes	Regulator	Commitment, Differentiation	[114]
Zfp217	Promotes	Regulator	Commitment, Differentiation	[104,105,106]
Zfp395	Promotes	Regulator	Commitment, Differentiation	[107]
Zfp423	Promotes	Marker	Commitment	[109]
		Regulator	Commitment	[108]
Zfp467	Promotes	Regulator	Commitment, Differentiation	[111,123,124]
Zfp521	Inhibits	Regulator	Commitment	[113]
WISP2	Inhibits	Regulator	Commitment, Differentiation	[122]

## 3. WAT Transcription

### 3.1. Mitotic Clonal Expansion (MCE)

Adipocyte differentiation is a highly strategic process that involves many transcription factors (TFs) and co-factors. Most of the established knowledge concerning this process has been acquired through various in vitro models, using either 3T3-L1 preadipocytes or primary preadipocytes isolated from adipose tissue [175]. It is widely accepted that PPARγ and C/EBPα serve as the principal transcriptional regulators of terminal adipocyte differentiation [176]. However, before the regulation and/or maintenance of PPARγ and C/EBPα, a series of events must occur, including: (i) cell commitment to the lineage (ii) preadipocyte growth arrest, (iii) MCE, (iv) early differentiation, and (v) late (terminal) differentiation (Figure 1A) [177,178]. 

In cell culture, preadipocytes are typically grown to confluence and become growth arrested due to cell contact–contact inhibition [179]. Synthetic stimulation (dexamethasone, insulin, and/or intracellular cAMP enhancers) induces growth-arrested preadipocytes to re-enter the cell cycle and undergo a couple more rounds of mitosis (e.g., MCE) as an essential prerequisite of terminal adipocyte differentiation, according to the field’s majority [157]. As such, disrupting cell cycle progression using various inhibitors including aphidicolin [180], roscovitine [181], and rapamycin [182] was found to interfere with adipocyte differentiation. Additionally, blocking caplain, an indispensable calcium-activated protease involved in MCE, with N-acetyl-Leu-Leu-norleucinal (ALLN) was shown to impair adipocyte differentiation when added 12 or 24 hours-post induction of differentiation [183]. On the other hand, others have reported that blocking MCE has a minimal or no effect on progression of differentiation. For example, after inhibiting MCE with PD98059 or U0126 (inhibitors of mitogen-activated protein kinase/extracellular signal-regulated kinase kinase 1) [184,185], adipocyte differentiation was not found to be impaired. Meanwhile, Tang et al. reported that PD98059 at 20 or 50 μΜ delayed MCE but did not block the process overall [181].

Regardless, upon the commencement of MCE, G1/S transition and DNA synthesis are reactivated, as evidenced by the activities of cell cycle regulators such as cyclins, cyclin-dependent kinases (cdk), and cdk inhibitors (CKIs) [186,187], as well as the renowned retinoblastoma (Rb)/E2F pathway [180,188]. Rb remains bound to E2F, obstructing its DNA binding capacity until successive phosphorylation by Cdk4/cyclin D and Cdk2/cyclin E is completed. Hyperphosphorylated Rb releases E2F, thus allowing for the transcriptional regulation of genes pertinent to adipocyte differentiation [181,189,190]. Members of the E2F family have been shown to act as either promoters or repressors of MCE. For instance, PPARγ expression was demonstrated to be induced by E2F1 during clonal expansion while, conversely, repressed during terminal differentiation by E2F4 [191].

Other factors such as sirtuin (SIRT) 6 have been noted to work upstream of a cascade that permits the phosphorylation and subsequent degradation of the cyclin-dependent kinase inhibitor, p27, a proposed requirement for MCE induction [192]. In support, an earlier study found that calpain-mediated p27 degradation was indeed necessary for the re-entry of preadipocytes into the cell cycle. Notably, inhibition of calpain blocked p27 degradation and halted Rb phosphorylation, both contributing to the overall prevention of adipocyte differentiation. However, interestingly, the expression of immediate early genes such as C/EBPβ and C/EBPδ remained unaltered [183]. Similarly, the induction of p21 expression, another CKI, via cyanidin-3-O-glucoside (C3G) exposure reduces MCE and arrests cells in the G0/G1 phase [193] (Figure 1C). 

While both aforementioned C/EBP members are known for regulating the expression of terminal differentiation factors, [194,195] they have also been implicated in playing a role at earlier stages of differentiation. Ex vivo evidence has reported that C/EBPβ null mouse embryonic fibroblasts lose their ability to undergo MCE or complete adipocyte differentiation [181]. Knockdown (KD) of C/EBPδ was found to downregulate the expression of different genes concurrently involved in MCE, including both factor for adipocyte differentiation 49 (fad49) and C/EBPβ [196]. In fact, it was previously observed that fad49 KD resulted in MCE arrest and reduced expression of C/EBPβ and C/EBPδ [197]. 

Genes associated with fat mass expansion and body weight regulation, such as the fat mass and obesity-associated gene, have been shown to positively regulate MCE by enhancing the expression of the pro-adipogenic RUNX1T1-S and stimulating the expression of D-type cyclins [198]. Proteome profiling identified pyruvate kinase M2 (PKM2) as another potential C/EBPβ target gene and MCE regulator. While PKM2 expression was high during early phases of differentiation, PKM2 KD compromised MCE [199]. More recently, Chen and colleagues revealed the involvement of a chromatin-associated protein, high-mobility group box 2 (HMGB2), which functions upstream of C/EBPβ during MCE. HMGB2 was shown to bind to the C/EBPβ promoter and enhance its expression and adipocyte differentiation [200] (Figure 1C).

While these studies imply that C/EBPβ and C/EBPδ are almost indispensable to the adipogenic cascade, other transcription factors have been suggested to possibly compensate upon their absence. For instance, olf-1/early B-cell factor (O/E-1) belongs to a family of basic helix-loop-helix (bHLH) transcription factors and has been reported as a potent adipogenic stimulator of genes crucial for terminal adipocyte differentiation [201]. Although the significance of the O/E members (e.g., O/E-1, -2, and -3) was originally attributed to lymphocyte and neuronal development, their expression has also been confirmed in adipose tissue and 3T3-L1 preadipocytes. In fact, expressions of both Ebf1 and 2 were demonstrated to precede the expression of the **master** transcription factors and exhibited an early expression pattern similar to that of C/EBPβ and C/EBPδ, suggesting they may also participate as early initiating factors [202]. The coordination of these various factors ultimately converges to maintain two of the most essential molecular players of adipocyte differentiation, PPARγ and C/EBPα. 

### 3.2. PPARγ 

The *PPARG* gene encodes for two primary isoforms, *PPARG1* (γ1) and *PPARG2* (γ2). PPARγ’s initial attribution to adipocyte development was observed by studies characterizing the transcriptional control of the tissue-specific aP2 gene [203,204]. PPARγ, mainly expressed in adipose tissue, functions as a nuclear hormone receptor and responds to a variety of lipid-based activators [205]. Early studies investigating the regulatory role of PPARγ determined its adipogenic propensity, whereby ectopic expression potently stimulated adipocyte differentiation in both fibroblasts and myoblasts [206,207]. 

Additionally, it was suggested that PPARγ2 has a more profound effect than its related isoform, PPARγ1 [208]. This inclination has been proposed due to its increased ability to bind to the DRIP/tartrate-resistant acid phosphatase (TRAP) system, a multi-subunit complex involved in adipogenic differentiation, among several other processes [209,210,211]. TRAP220, a subunit of the TRAP complex, plays a vital coactivating role in PPARγ2-stimulated adipose development. As such, *Trap220^−/−^* fibroblasts lacked the ability to fully differentiate into adipocytes under PPARγ2 stimulation, while ectopic expression of TRAP220 restored morphological defects as well as several key endogenous adipogenic markers including *Fabp4*, *Slc2a4*, *Pparg*, and *Cebpa* [211].

Due to the challenge of embryonic lethality presented by PPARγ-null mice, the majority of loss-of-function experiments are heavily reliant on results obtained in heterozygous mutants [212,213,214]. The generation of chimeric mice revealed the necessity of PPARγ, as *Pparg*-null cells lack in contribution to adequate fat formation [213]. Similarly, one viable mouse lacking all forms of adipose tissues, generated with the use of a tetraploid embryonic approach, convinced investigators to conclude that PPARγ critically controls adipose tissue development in vivo [212]. 

PPARγ has also been linked to lipid metabolism and insulin sensitivity, both attributes of adipose tissue core metabolism [215]. For instance, *Pparg^+/−^* mice were found to be more insulin-sensitive and protected against age-associated insulin resistance [216,217]. On the other hand, adipose-specific *Pparg* knockout mice suffer from lipodystrophy and hyperlipidemia [218]. Proceeding reports have shown that these mice experience hypotension, hepatic steatosis, and abnormally enlarged pancreatic islets, metabolic consequences related to the disturbance of proper fat formation [219,220,221,222]. Furthermore, studies examining *PPARG* mutations in human adipose have revealed several associated metabolic disturbances such as lipodystrophy, diabetes, hypertension, and insulin resistance [223,224]. Importantly, these findings highlight the clinical relevance of properly maintaining the role of PPARγ in WAT. 

### 3.3. C/EBPα

Likewise, C/EBPα also plays a prominent role in adipocyte differentiation [225]. An early study investigating the transcriptional basis of adipocyte development found that gene transfection of *Cebpa* in 3T3-L1 fibroblasts led to the development of an adipocyte morphology. However, 3T3-L1 cells with excessive c-Myc expression were unable to differentiate nor promote the expression of adipogenic genes, even though c-Myc is a pivotal regulator of several biological processes such as differentiation and proliferation, [226,227]. While some [228,229,230] propose that C/EBPα is sufficient to initiate and direct the adipocyte differentiation program of 3T3-L1 fibroblasts, others argue that C/EBPα works most efficiently in concert with additional regulators, namely PPARγ. Although it is well-established that PPARγ is a key activator of adipocyte differentiation, C/EBPα is able to ally its efforts with PPARγ to promote differentiation [194,231,232]. In fact, co-expression of these factors demonstrated a synergistic activation of various genes involved in adipocyte metabolism, including *Fabp4*, Solute carrier family 2, facilitated glucose transporter member 4 (*Slc2a4* a.k.a. *Glut4)*, and *Lep* [231,233]. PPARγ and C/EBPα foster a feedforward system by cross regulating each other’s expression to maintain high levels of PPARγ, a characteristic feature of differentiated adipocytes [231]. It has also been shown that C/EBPα regulates the expression of transcription factors related to adipocyte metabolism, including SIRT1, a notable regulator of lipolysis and insulin sensitivity [234,235,236]. 

The generation of C/EBPα knockout mice unexpectedly resulted in neonatal lethality as animals succumbed to severe hypoglycemia within eight hours of birth. This predicament was partially overcome by an incremental administration of glucose postpartum. However, surviving *Cebpα^−/−^* mice were unable to accumulate lipids in both white and brown depots, although several key adipocyte markers in BAT remained unaltered [237,238]. 

In order to further investigate C/EBPα’s regulatory role in adipose development in vivo, Linhart et al. created a transgenic model to counter the lethargic tendency presented by *Cebpα^−/−^* mice and improved their survival rate by 2.7-fold. These transgenic mice exhibited the absence of several white adipose depots, including subcutaneous, perirenal, and epididymal fat, concomitantly with elevated serum lipid levels. Interestingly, BAT and adipocytes found within the mammary tissues were found to be only slightly hypertrophied, yet were present and exhibited a normal morphology, respectively [239]. 

Moreover, conditional knockout mice (*Cebpa^Δ/−^*), resulting in a near-total postnatal ablation of C/EBPα in several tissues including white (~89%) and brown (~80%) adipose, demonstrated various altered metabolic responses including hypophagia, hypoglycemia, hypoinsulinemia, weight loss, and development of hepatic steatosis. *Cebpa^Δ/−^* mice were also found to lose a considerable amount of triglyceride from white, but not brown, depots, as evident by lipodystrophy within the epididymal, subcutaneous, and periovarian fat pads [240]. In an adipose-specific transgenic model targeting C/EBP members, morphological signs of white subcutaneous lipodystrophy as well as an altered expression of several well-characterized gene targets of C/EBPα in inguinal fat were evident [241]. Therefore, these studies thoroughly indicate that C/EBPα, in addition to PPARγ, is indeed a critical coregulator of adipocyte differentiation.

### 3.4. Additional Modulators

While most of the emphasis is dedicated to the factors described above, it is recognized that there are a variety of different transcription factors which play significant roles in the regulation of adipocyte differentiation (Figure 1D). For instance, members of the Krüppel-like factor (KLF) family have been reported to either inhibit or promote differentiation, with KLF15 reported as the founding member. KLF15 has been demonstrated to transcriptionally regulate the adipogenic cascade through a direct modulation of PPARγ [242]. In addition, upstream modulation of this factor has been shown to regulate adipocyte differentiation. For example, the glucocorticoid receptor was demonstrated to directly target KLF15 and promote its expression through DNA-binding interactions, thus promoting adipocyte differentiation [243]. 

Conversely, c-Jun was shown to downregulate KLF15 expression and consequently suppress adipocyte differentiation [244]. Other KLF family members identified as pro-adipogenic include KLF4, 5, 6, 8, and 9. Briefly, KLF members 4, 5, 6, 8, and 9 promote early- (C/EBPβ) and late- (PPARγ, C/EBPα) stage differentiation markers, while KLF6 also promotes differentiation by repressing Pref-1 [245,246]. 

KLFs members demonstrating anti-adipogenic activity include members such as KLF2, 3, and 7. Protein levels and the promoter activity of KLF2 share an inverse relationship with the differentiation status of preadipocytes. It has been proposed that KLF2 inhibits adipocyte differentiation by modulating the expression of PPARγ and partially restoring the expression of Pref-1 [247,248]. Comparatively, KLF3 negatively controls differentiation by exerting its inhibitory effects on C/EBPα, while KLF7 suppresses the expression of several hallmark proteins, namely C/EBPα, PPARγ, Adipsin, and aP2 [249,250]. Once again, adipocyte differentiation is regulated by a diverse collection of transcription factors, not just by one family. Additional modulators of white adipose differentiation are briefly summarized in Table 2 and reviewed in detail elsewhere [176,251,252,253,254]. 

The Wnt family proteins are of particular interest due to their pleiotropic roles in numerous biological processes relevant to the whole process of adipogenesis, such as embryonic development, cell proliferation, and tissue homeostasis. These highly conserved proteins, found in most multicellular organisms, exert diverse regulatory influences on adipocyte differentiation with some members promoting and others inhibiting the differentiation process, depending on the experimental model [255,256,257]. The role of other members, however, remains controversial, as Wnt 5a has been shown to both positively and negatively affect adipocyte differentiation [258,259]. The reasons for these conflicting findings are not yet fully understood. However, it is important to note that the regulatory effects of Wnt proteins can be mediated through both canonical and non-canonical signaling pathways.

The canonical Wnt signaling pathway mediated through β-catenin, impedes adipogenesis through several mechanisms that repress the expression and transcriptional activity of the master regulators, C/EBPα/β and PPARγ [255,260,261], justifying the widespread interest in this pathway as a potential target for anti-obesity strategies. However, not all Wnt ligands function identically. For instance, Wnt-1 and Wnt10b inhibit adipocyte differentiation by stabilizing β-catenin, thereby preventing the differentiation of preadipocytes through the inhibition of PPARγ and C/EBPα/β [255,260] (Table 2; Figure 1D). In contrast, Wnt5b promotes adipogenesis by inhibiting the canonical pathway, reducing β-catenin nuclear translocation, and downregulating genes such as IGF-1, VEGF-C, and WISP-1 [262]. Similarly, Wnt ligands can also influence non-canonical pathways, although the effects on differentiation can, again, vary depending on the experimental model, dosage, and duration of treatment. For example, in mesenchymal stem cells and committed preadipocytes, low concentrations of Wnt5a inhibit adipocyte differentiation, while high concentrations promote it by interfering with β-catenin activity [259,263]. 

**Table 2:** Select Proteins Involved in the Regulation of Terminal Adipocyte Differentiation. For each protein, the target factor mediating its role in adipogenic differentiation is shown.

Transcription Factors/Regulators	Primary Effect	Target(s)	Reference(s)
ATG4B	Inhibits	KLF2,3	[264]
C/EBPα	Promotes	Sirt1	[236]
C/EBPβ	Promotes	PPARγ, C/EBPα	[225,238]
C/EBPδ			
CHOP10	Inhibits	C/EBPβ	[265]
CREB	Promotes	C/EBPβ	[266]
EBF1	Promotes	PPARγ, C/EBPα	[202]
GATA2/3	Inhibits	PPARγ	[267]
GLP-1	Promotes	PPARγ, C/EBPα	[268]
Krox20	Promotes	C/EBPβ	[269]
LXR	Promotes	PPARγ	[270]
Sirt1	Inhibits	PPARγ	[234,271]
Sox6	Promotes	PPARγ, C/EBPα, β, δ	[272]
SREBP1c/ADD1	Promotes	PPARγ	[273]
TGF-β	Inhibits	C/EBPβ, δ	[274]
Wnt1	Inhibits	PPARγ, C/EBPα	[275]
Wnt3a			[276]
Wnt4	Promotes		[258,268]
Wnt5a	Promotes		[258]
Wnt5a	Inhibits		[259]
Wnt5b	Promotes		[262,277]
Wnt6	Inhibits		[278]
Wnt8			[279]
Wnt10a/b			[278,280]
XBP1	Promotes	PPARγ, C/EBPα	[281,282]
Zfp423	Promotes	PPARγ	[108]
Zfp638	Promotes	C/EBPβ, δ	[283]

## 4. Future Directions 

Over the years, WAT has gained better recognition, particularly for its roles in energy homeostasis and endocrine function [284,285]. This growing appreciation is further underscored by studies highlighting the metabolic consequences of conditions like lipoatrophy, where WAT is absent [241,286]. However, in regions like the United States, where an excessive accumulation of WAT is highly prevalent (e.g., obesity), efforts have predominantly been focused on strategies to reduce WAT, not promote or maintain it [287]. Fortunately, adipose tissue, whether WAT or BAT, is known to exhibit a high degree of plasticity and this could be used therapeutically for a variety of diseases and metabolic disorders. Moreover, this inherent feature may be exploited largely from stem cell commitment to terminal differentiation. 

As discussed previously, BMP4 is a key signaling factor known to commit stem cells to the adipogenic lineage [288]. However, additional studies suggest that BMP4 may actually play a dual role in the process of adipocyte development, guiding not just the initial commitment of stem cells but also the phenotypic fate of preadipocytes upon differentiation [289,290]. For example, Gustafson et al. found that the maintenance of white adipose tissue determination is due to an integral feedback loop between BMP4 signaling and BMP4 antagonists secreted by mature adipocytes and preadipocytes, respectively [289]. This interplay also facilitates the hyperplasic expansion of WAT. The secretion of BMP4 by mature white adipocytes first commits mesenchymal stems cells to the adipogenic lineage and then BMP4 antagonists later control the fate of the preadipocytes towards a white lineage [289]. Interestingly, research has also shown that BMP4 alone or the silencing of BMP4 antagonists can promote a brown-like phenotype in fully differentiated white adipocytes [289,291], which presents a promising therapeutic avenue for mitigating metabolic complications associated with weight regain. Notably, these findings suggest a broader role for BMP4 in adipogenesis, extending beyond differentiation to encompass the entire process. Thus, this warrants further investigation to elucidate its precise mechanisms and therapeutic potential.

Furthermore, weight regain is a common cause for concern due to the negative metabolic outcomes that were intended to be avoided with the initial weight loss. Current explanations for the propensity for weight regain include the involvement of altered secretion profiles of adipocytes and gut hormones, extracellular matrix remodeling, downregulated lipolysis, and inflammatory signaling [292,293]. Further elucidation of the molecular mechanisms of weight regain in white adipose tissue suggests that weight regain is primarily influenced by the refilling of established white adipocytes rather than increased recruitment and differentiation of preadipocytes [292]. With further research, BMP4 could exist as a promising target to reduce the consequences of white adipocyte refilling by establishing a beige phenotype that has been shown to increase energy expenditure and improve insulin sensitivity [294]. Likewise, the potential of BMP4 to commit cells to the adipogenic lineage could be used to remodel WAT by introducing preadipocytes to the tissues that are unaffected by the adipocyte shrinkage from weight loss. 

Traditionally, adipocyte differentiation was thought to be a permanent end, where mature adipocytes lose their ability to proliferate and would store excess energy until their death. However, in the late 1980s Sugihara et al. first introduced the ceiling culture technique and observed a unique phenomenon: mature adipocytes revert into fibroblastic progenitor-like cells with proliferative capabilities [295,296]. This progeny of revert cells would later be widely referred to as dedifferentiated fat (DFAT). In the process of dedifferentiation, adipocytes alter their morphology from uniocular, lipid-loaded, rounded cells to spindle-shaped cells devoid of lipid droplets. Notably, DFAT cells display a significant downregulation of adipogenic markers such as PPARγ, aP2, perilipin 1, and adiponectin [297,298] and express several cell surface markers associated with “stemness”. In addition, DFAT cells also possess the ability to re-differentiate or even transdifferentiate into other mesenchymal types [297,299,300,301].

Several studies have been carried out to understand the mechanism of the adipocyte dedifferentiation. Major signaling pathways such as Wnt/β-catenin, TGF-β1, and Notch have all been identified to play a role [299,302]. For example, the activation of Wnt signaling by the Wnt3a ligand was shown to upregulate undifferentiated gene markers while concurrently suppressing adipogenic markers (PPARγ, C/EBPα, FABP4, adiponectin, and GLUT4), suggesting a role in dedifferentiation [302]. TGF-β1 has been proposed as a regulator of adipocyte dedifferentiation by stimulating SMAD 2/3 phosphorylation and the expression of several collagen genes [303]. However, its sufficiency in inducing this process remains contested, with evidence suggesting the requirement of PPARγ downregulation [304]. Furthermore, despite the fact that Notch signaling has been demonstrated to promote cell dedifferentiation, its associated metabolic outcomes remain controversial. In some mice studies, Notch activation may have promoted adipocyte dedifferentiation or blocked WAT expansion, but activation also conferred insulin resistance and tumorigenic transformation [305,306]. On the other hand, Siouti and colleagues recently revealed a protective role of adipose tissue Notch signaling. Indeed, the authors concluded that a combined abrogation of Notch 1 and 2 receptors results in the development of AT inflammation and metabolic dysregulation [307]. 

Due to their abundance and low immunogenicity, DFAT cells are promising candidates for various therapeutic applications [300], however, careful consideration of potential risks and limitations is essential. In addition to the potential metabolic issues indicated above, in vitro studies involving DFAT cells often encounter technical challenges. For example, the commonly employed ceiling culture method for investigating cell dedifferentiation is susceptible to cross-contamination due to the unintended “dragging” effect or the potential co-isolation of cell types of a similar buoyancy to DFAT cells [308].

## 5. Conclusions

Despite its initial atrocious façade, white adipose tissue is an essential component of human health, just not in excess. Although the main objective of any review is the breadth of coverage, covering a topic as dynamic as WAT is considerably challenging. Therefore in this review, various themes have been emphasized: First, white adipose tissue constitutes more than just the main parenchymal cells of the organ. As well, new and improved technologies are stealthily advancing our understanding of each cell type’s (1) identity and (2) contribution to WAT development. Second, it remains clear that adipocyte differentiation involves a complex cascade of various transcription factors, co-factors, and signaling pathways that lend guidance from commitment through terminal differentiation. However, equating adipocyte differentiation to adipogenesis can no longer be justified as the latter scales far beyond the differentiation process. Inevitably, while many questions remain, understanding the roles or underlying biological mechanisms of factors involved in the regulation of preadipocyte commitment and differentiation could help to design better therapeutic strategies against diseases associated with adipose tissues. 

## Data Availability

No new data were created or analyzed in this study. Data sharing is not applicable to this article.

## Abbreviations

Adipogenesis-regulatory (Areg); Adipose mesenchymal stem cells (Ad-MSCs); Adipose precursor cells (APCs); Adipose progenitor (AP); Adipose stem cells (ASCs); Adipose stem and progenitor cells (ASPCs); Adipose tissue (AT); Adipose-derived stromal (ADSCs); B-cell lymphoma 6 (Bcl6); Basic helix-loop-helix (bHLH); Betaine-homocysteine S-methyltransferase (BHMT); Bone marrow mesenchymal stem cells (BMSCs); Bone morphogenetic proteins (BMPs); Brown adipose tissue (BAT); cyanidin-3-O-glucoside (C3G); Cardiovascular disease (CVD); CCAAT-enhancer-binding protein (C/EBP); Cdk inhibitors (CKIs); Cyclic AMP (cAMP); Cyclin-dependent kinases (cdks); de novo lipogenesis (DNL); Dedifferentiated fat (DFAT); Delta-like homolog–1 (Dlk1); Dipeptidyl peptidase–4 (DPP4+); DRIP/tartrate-resistant acid phosphatase (TRAP); Early B cell factor 1 (Ebf1); Factor for adipocyte differentiation 49 (fad49); Fatty acid binding protein 4 (Fabp4); Fibro/adipogenic progenitors (FAPs); Glucose transporter 4 (GLUT4); High-mobility group box 2 (HMGB2); Human umbilical vein endothelial cells (HUVECs); Insulin receptor substrate 1 (IRS1); Insulin resistance (IR); Intercellular adhesion molecule–1 (ICAM1; Knockdown (KD); Knockout (KO); Krüppel-like factor (KLF); Metabolically healthy obesity (MHO); Metabolically unhealthy obesity (MUO); N-acetyl-Leu-Leu-norleucinal (ALLN); Olf-1/early B-cell factor (O/E-1); p38 mitogen-activated protein kinase (MAPK; Peroxisome proliferator-activated receptor gamma (PPARγ); Pink adipocytes (PATs); Platelet-derived growth factor receptor alpha (Pdgfra); PPARγ coactivator-1 alpha (PGC-1α); PR domain containing 16 (PRDM16); Pyruvate kinase M2 (PKM2); Retinoblastoma protein (pRb); Single cell RNA sequencing (scRNA-seq); Sirtuin (SIRT); Smooth muscle actin (SMA+); Solute carrier family 2, facilitated glucose transporter member 4 (Slc2a4); SRY-box transcription factor 9 (Sox9); Stromal vascular fraction (SVF); Subcutaneous (SAT); Suppressor of Mothers against Decapentaplegic (SMAD); Sympathetic nervous system (SNS); Transcription factor 7-like 1 (TCF7L1); Transcription factors (TFs); Transforming growth factor beta-1 (TGF-β1); Triacylglycerol (TAG); Type 2 diabetes mellitus (T2DM); Uncoupling protien 1 (UCP1); Visceral (VAT); White adipose tissue (WAT); WNT1 inducible signaling pathway protein 2 (WISP2); Yellow adipose tissue (YAT); Zinc finger E-box-binding homeobox 1 (ZEB1); Zinc finger proteins (Zfp).
